# Global Guidance for Local Generalization in Model Checking

**DOI:** 10.1007/978-3-030-53291-8_7

**Published:** 2020-06-16

**Authors:** Hari Govind Vediramana Krishnan, YuTing Chen, Sharon Shoham, Arie Gurfinkel

**Affiliations:** 8 Microsoft Research Lab, Redmond, WA USA; 9 University of Southern California, Los Angeles, CA USA; 10 University of Waterloo, Waterloo, Canada; 11 Chalmers University of Technology, Gothenburg, Sweden; 12 Tel Aviv University, Tel Aviv, Israel

## Abstract

SMT-based model checkers, especially IC3-style ones, are currently the most effective techniques for verification of infinite state systems. They infer *global* inductive invariants via *local* reasoning about a single step of the transition relation of a system, while employing SMT-based procedures, such as interpolation, to mitigate the limitations of local reasoning and allow for better generalization. Unfortunately, these mitigations intertwine model checking with heuristics of the underlying SMT-solver, negatively affecting stability of model checking.

In this paper, we propose to tackle the limitations of locality in a systematic manner. We introduce explicit *global guidance* into the local reasoning performed by IC3-style algorithms. To this end, we extend the SMT-IC3 paradigm with three novel rules, designed to mitigate fundamental sources of failure that stem from locality. We instantiate these rules for the theory of Linear Integer Arithmetic and implement them on top of Spacer solver in Z3. Our empirical results show that GSpacer, Spacer extended with global guidance, is significantly more effective than both Spacer and sole global reasoning, and, furthermore, is insensitive to interpolation.

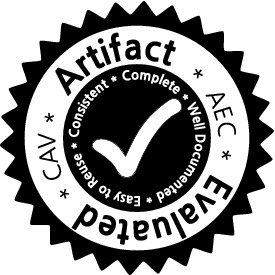



## Introduction

SMT-based Model Checking algorithms that combine SMT-based search for bounded counterexamples with interpolation-based search for inductive invariants are currently the most effective techniques for verification of infinite state systems. They are widely applicable, including for verification of synchronous systems, protocols, parameterized systems, and software.

The Achilles heel of these approaches is the mismatch between the *local* reasoning used to establish absence of bounded counterexamples and a *global* reason for absence of unbounded counterexamples (i.e., existence of an inductive invariant). This is particularly apparent in IC3-style algorithms [7], such as Spacer [18]. IC3-style algorithms establish bounded safety by repeatedly computing predecessors of error (or bad) states, blocking them by local reasoning about a single step of the transition relation of the system, and, later, using the resulting *lemmas* to construct a candidate inductive invariant for the global safety proof. The whole process is driven by the choice of local lemmas. Good lemmas lead to quick convergence, bad lemmas make even simple-looking problems difficult to solve.

The effect of local reasoning is somewhat mitigated by the use of interpolation in lemma construction. In addition to the usual inductive generalization by dropping literals from a blocked bad state, interpolation is used to further generalize the blocked state using theory-aware reasoning. For example, when blocking a bad state $x = 1 \wedge y = 1$, inductive generalization would infer a sub-clause of $x \ne 1 \vee y \ne 1$ as a lemma, while interpolation might infer $x \ne y$ – a predicate that might be required for the inductive invariant. Spacer, that is based on this idea, is extremely effective, as demonstrated by its performance in recent CHC-COMP competitions [10]. The downside, however, is that the approach leads to a highly unstable procedure that is extremely sensitive to syntactic changes in the system description, changes in interpolation algorithms, and any algorithmic changes in the underlying SMT-solver.

An alternative approach, often called *invariant inference*, is to focus on the global safety proof, i.e., an inductive invariant. This has long been advocated by such approaches as Houdini [15], and, more recently, by a variety of machine-learning inspired techniques, e.g., FreqHorn [14], LinearArbitrary [28], and ICE-DT [16]. The key idea is to iteratively generate positive (i.e., reachable states) and negative (i.e., states that reach an error) examples and to compute a candidate invariant that separates these two sets. The reasoning is more focused towards the invariant, and, the search is restricted by either predicates, templates, grammars, or some combination. Invariant inference approaches are particularly good at finding simple inductive invariants. However, they do not generalize well to a wide variety of problems. In practice, they are often used to complement other SMT-based techniques.

In this paper, we present a novel approach that extends, what we call, *local reasoning* of IC3-style algorithms with *global guidance* inspired by the invariant inference algorithms described above. Our main insight is that the set of lemmas maintained by IC3-style algorithms hint towards a potential global proof. However, these hints are lost in existing approaches. We observe that letting the current set of lemmas, that represent candidate global invariants, guide local reasoning by introducing new lemmas and states to be blocked is often sufficient to direct IC3 towards a better global proof.

We present and implement our results in the context of Spacer—a solver for Constrained Horn Clauses (CHC)—implemented in the Z3 SMT-solver [13]. Spacer is used by multiple software model checking tools, performed remarkably well in CHC-COMP competitions [10], and is open-sourced. However, our results are fundamental and apply to any other IC3-style algorithm. While our implementation works with arbitrary CHC instances, we simplify the presentation by focusing on infinite state model checking of transition systems.

**Fig. 1.**
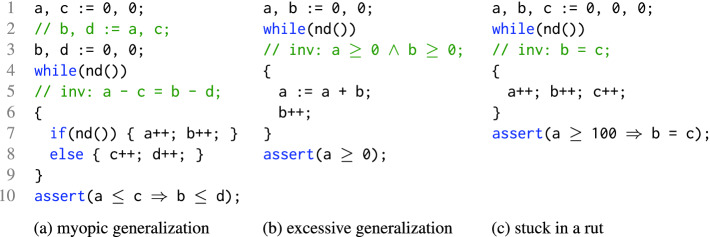
Verification tasks to illustrate sources of divergence for Spacer. The call *nd*() non-deterministically returns a Boolean value.

We illustrate the pitfalls of local reasoning using three examples shown in Fig. 1. All three examples are small, simple, and have simple inductive invariants. All three are challenging for Spacer. Where these examples are based on Spacer-specific design choices, each exhibits a fundamental deficiency that stems from local reasoning. We believe they can be adapted for any other IC3-style verification algorithm. The examples assume basic familiarity with the IC3 paradigm. Readers who are not familiar with it may find it useful to read the examples after reading Sect. 2.

*Myopic Generalization.* Spacer diverges on the example in Fig. 1(a) by iteratively learning lemmas of the form $( a - c \le k ) \Rightarrow ( b - d \le k )$ for different values of *k*, where *a*, *b*, *c*, *d* are the program variables. These lemmas establish that there are no counterexamples of longer and longer lengths. However, the process never converges to the desired lemma $( a - c ) \le ( b - d )$, which excludes counterexamples of any length. The lemmas are discovered using interpolation, based on proofs found by the SMT-solver. A close examination of the corresponding proofs shows that the relationship between $(a-c)$ and $(b-d)$ does not appear in the proofs, making it impossible to find the desired lemma by tweaking local interpolation reasoning. On the other hand, looking at the global proof (i.e., the set of lemmas discovered to refute a bounded counterexample), it is almost obvious that $(a-c) \le (b-d)$ is an interesting generalization to try. Amusingly, a small, syntactic, but semantic preserving change of swapping line 2 for line 3 in Fig. 1(a) changes the SMT-solver proofs, affects local interpolation, and makes the instance trivial for Spacer.

*Excessive (Predecessor) Generalization.* Spacer diverges on the example in Fig. 1(b) by computing an infinite sequence of lemmas of the form $a + k_1 \times b \ge k_2$, where *a* and *b* are program variables, and $k_1$ and $k_2$ are integers. The root cause is excessive generalization in predecessor computation. The $Bad$ states are $a < 0$, and their predecessors are states such as $(a = 1 \wedge b = -10)$, $(a = 2 \wedge b = -10)$, etc., or, more generally, regions $(a + b < 0)$, $(a + 2b < -1)$, etc. Spacer always attempts to compute the most general predecessor states. This is the best local strategy, but blocking these regions by learning their negation leads to the aforementioned lemmas. According to the global proof these lemmas do not converge to a linear invariant. An alternative strategy that under-approximates the problematic regions by (numerically) simpler regions and, as a result, learns simpler lemmas is desired (and is effective on this example). For example, region $a + 3b \le -4$ can be under-approximated by $a \le 32 \wedge b \le -12$, eventually leading to a lemma $b \ge 0$, that is a part of the final invariant: $(a \ge 0 \wedge b \ge 0)$.

*Stuck in a Rut.* Finally, Spacer converges on the example in Fig. 1(c), but only after unrolling the system for 100 iterations. During the first 100 iterations, Spacer learns that program states with $$(a \ge 100 \wedge b \ne c)$$ are not reachable because *a* is bounded by 1 in the first iteration, by 2 in the second, and so on. In each iteration, the global proof is updated by replacing a lemma of the form $$a < k$$ by lemma of the form $$ a < (k+1)$$ for different values of *k*. Again, the strategy is good locally – total number of lemmas does not grow and the bounded proof is improved. Yet, globally, it is clear that no progress is made since the same set of bad states are blocked again and again in slightly different ways. An alternative strategy is to abstract the literal $$a \ge 100$$ from the formula that represents the bad states, and, instead, conjecture that no states in $$b \ne c$$ are reachable.

*Our Approach: Global Guidance.* As shown in the examples above, in all the cases that Spacer diverges, the missteps are not obvious locally, but are clear when the overall proof is considered. We propose three new rules, Subsume, Concretize, and, Conjecture, that provide global guidance, by considering existing lemmas, to mitigate the problems illustrated above. Subsume introduces a lemma that generalizes existing ones, Concretize under-approximates partially-blocked predecessors to focus on repeatedly unblocked regions, and Conjecture over-approximates a predecessor by abstracting away regions that are repeatedly blocked. The rules are generic, and apply to arbitrary SMT theories. Furthermore, we propose an efficient instantiation of the rules for the theory Linear Integer Arithmetic.

We have implemented the new strategy, called GSpacer, in Spacer and compared it to the original implementation of Spacer. We show that GSpacer outperforms Spacer in benchmarks from CHC-COMP 2018 and 2019. More significantly, we show that the performance is independent of interpolation. While Spacer is highly dependent on interpolation parameters, and performs poorly when interpolation is disabled, the results of GSpacer are virtually unaffected by interpolation. We also compare GSpacer to LinearArbitrary [28], a tool that *infers invariants* using global reasoning. GSpacer outperforms LinearArbitrary on the benchmarks from [28]. These results indicate that global guidance mitigates the shortcomings of local reasoning.

The rest of the paper is structured as follows. Sect. 2 presents the necessary background. Sect. 3 introduces our *global guidance* as a set of abstract inference rules. Sect. 4 describes an instantiation of the rules to Linear Integer Arithmetic (LIA). Sect. 5 presents our empirical evaluation. Finally, Sect. 7 describes related work and concludes the paper.

## Background

*Logic.* We consider first order logic modulo theories, and adopt the standard notation and terminology. A first-order language modulo theory $\mathcal {T}$ is defined over a signature $\varSigma$ that consists of constant, function and predicate symbols, some of which may be *interpreted* by $\mathcal {T}$. As always, *terms* are constant symbols, variables, or function symbols applied to terms; *atoms* are predicate symbols applied to terms; *literals* are atoms or their negations; *cubes* are conjunctions of literals; and *clauses* are disjunctions of literals. Unless otherwise stated, we only consider *closed* formulas (i.e., formulas without any free variables). As usual, we use sets of formulas and their conjunctions interchangeably.

*MBP.* Given a set of constants $\textit{\textbf{v}}$, a formula $\varphi$ and a model $M \models \varphi$, Model Based Projection (MBP) of $\varphi$ over the constants $\textit{\textbf{v}}$, denoted $\textsc {MBP}(\textit{\textbf{v}}, \varphi , M)$, computes a model-preserving under-approximation of $\varphi$ projected onto $\varSigma \setminus \textit{\textbf{v}}$. That is, $\textsc {MBP}(\textit{\textbf{v}}, \varphi , M)$ is a formula over $\varSigma \setminus \textit{\textbf{v}}$ such that $M \models \textsc {MBP}(\textit{\textbf{v}}, \varphi , M)$ and any model $M' \models \textsc {MBP}(\textit{\textbf{v}}, \varphi , M)$ can be extended to a model $M'' \models \varphi$ by providing an interpretation for $\textit{\textbf{v}}$. There are polynomial time algorithms for computing MBP in Linear Arithmetic [5, 18].

*Interpolation.* Given an unsatisfiable formula $A \wedge B$, an interpolant, denoted $\textsc {ITP}(A,B)$, is a formula *I* over the shared signature of *A* and *B* such that $A \Rightarrow I$ and $I \Rightarrow \lnot B$.

*Safety Problem.* A *transition system* is a pair $\langle Init , Tr \rangle$, where $Init$ is a formula over $\varSigma$ and $Tr$ is a formula over $\varSigma \cup \varSigma '$, where $\varSigma ' = \{s' \mid s \in \varSigma \}$.^1^ The states of the system correspond to structures over $\varSigma$, $Init$ represents the initial states and $Tr$ represents the transition relation, where $\varSigma$ is used to represent the pre-state of a transition, and $\varSigma '$ is used to represent the post-state. For a formula $\varphi$ over $\varSigma$, we denote by $\varphi '$ the formula obtained by substituting each $s \in \varSigma$ by $s' \in \varSigma '$. A *safety problem* is a triple $\langle Init , Tr , Bad \rangle$, where $\langle Init , Tr \rangle$ is a transition system and $Bad$ is a formula over $\varSigma$ representing a set of bad states.

The safety problem $$\langle Init , Tr , Bad \rangle $$ has a *counterexample of length k* if the following formula is satisfiable: $$ Init ^0 \wedge \bigwedge _{i=0}^{k-1} Tr ^i \wedge Bad ^k, $$ where $$\varphi ^i$$ is defined over $$\varSigma ^i = \{s^i \mid s \in \varSigma \}$$ (a copy of the signature used to represent the state of the system after the execution of *i* steps) and is obtained from $$\varphi $$ by substituting each $$s \in \varSigma $$ by $$s^i \in \varSigma ^i$$, and $$ Tr ^i$$ is obtained from $$ Tr $$ by substituting $$s \in \varSigma $$ by $$s^i \in \varSigma ^i$$ and $$s' \in \varSigma '$$ by $$s^{i+1} \in \varSigma ^{i+1}$$. The transition system is *safe* if the safety problem has no counterexample, of any length.

*Inductive Invariants.* An *inductive invariant* is a formula $Inv$ over $\varSigma$ such that (i) $Init \Rightarrow Inv$, (ii) $Inv \wedge Tr \Rightarrow Inv '$, and (iii) $Inv \Rightarrow \lnot Bad$. If such an inductive invariant exists, then the transition system is safe.

*Spacer.* The safety problem defined above is an instance of a more general problem, CHC-SAT, of satisfiability of Constrained Horn Clauses (CHC). Spacer is a semi-decision procedure for CHC-SAT. However, to simplify the presentation, we describe the algorithm only for the particular case of the safety problem. We stress that Spacer, as well as the developments of this paper, apply to the more general setting of CHCs (both linear and non-linear). We assume that the only uninterpreted symbols in $\varSigma$ are constant symbols, which we denote $\textit{\textbf{x}}$. Typically, these represent program variables. Without loss of generality, we assume that $Bad$ is a cube.
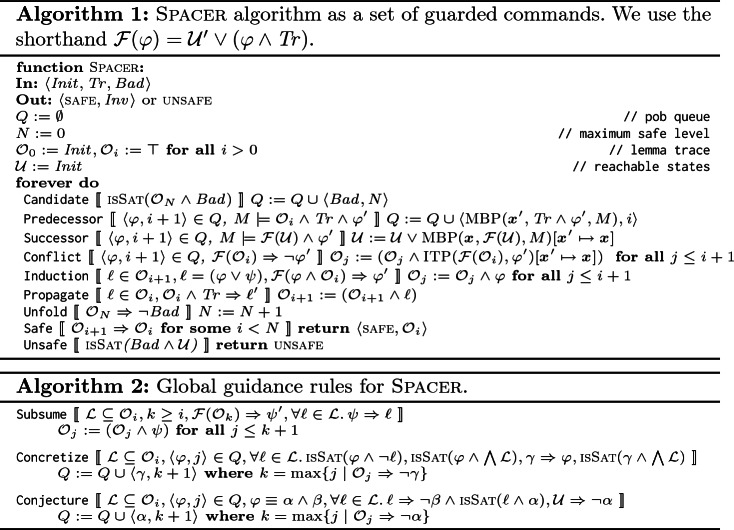


Algorithm 1 presents the key ingredients of Spacer as a set of guarded commands (or rules). It maintains the following. Current unrolling depth *N* at which a counterexample is searched (there are no counterexamples with depth less than *N*). A *trace* $\mathcal {O}= (\mathcal {O}_0, \mathcal {O}_1, \ldots )$ of *frames*, such that each frame $\mathcal {O}_i$ is a set of *lemmas*, and each lemma $\ell \in \mathcal {O}_i$ is a clause. A queue of *proof obligations* *Q*, where each proof obligation (pob) in *Q* is a pair $\langle \varphi , i \rangle$ of a cube $\varphi$ and a level number *i*, $0 \le i \le N$. An under-approximation $\mathcal {U}$ of reachable states. Intuitively, each frame $\mathcal {O}_i$ is a candidate inductive invariant s.t. $\mathcal {O}_i$ over-approximates states reachable up to *i* steps from $Init$. The latter is ensured since $\mathcal {O}_0 = Init$, the trace is monotone, i.e., $\mathcal {O}_{i+1} \subseteq \mathcal {O}_i$, and each frame is inductive *relative* to its previous one, i.e., $\mathcal {O}_i \wedge Tr \Rightarrow \mathcal {O}_{i+1}'$. Each pob $\langle \varphi , i \rangle$ in *Q* corresponds to a suffix of a potential counterexample that has to be blocked in $\mathcal {O}_i$, i.e., has to be proven unreachable in *i* steps.

The Candidate rule adds an initial pob $\langle Bad , N \rangle$ to the queue. If a pob $\langle \varphi , i \rangle$ cannot be blocked because $\varphi$ is reachable from frame $(i-1)$, the Predecessor rule generates a predecessor $\psi$ of $\varphi$ using MBP and adds $\langle \psi , i-1 \rangle$ to *Q*. The Successor rule updates the set of reachable states if the pob is reachable. If the pob is blocked, the Conflict rule strengthens the trace $\mathcal {O}$ by using interpolation to learn a new lemma $\ell$ that blocks the pob, i.e., $\ell$ implies $\lnot \varphi$. The Induction rule strengthens a lemma by inductive generalization and the Propagate rule pushes a lemma to a higher frame. If the $Bad$ state has been blocked at *N*, the Unfold rule increments the depth of unrolling *N*. In practice, the rules are scheduled to ensure progress towards finding a counterexample.

## Global Guidance of Local Proofs

As illustrated by the examples in Fig. 1, while Spacer is generally effective, its local reasoning is easily confused. The effectiveness is very dependent on the local computation of predecessors using model-based projection, and lemmas using interpolation. In this section, we extend Spacer with three additional *global* reasoning rules. The rules are inspired by the deficiencies illustrated by the motivating examples in Fig. 1. In this section, we present the rules abstractly, independent of any underlying theory, focusing on pre- and post-conditions. In Sect. 4, we specialize the rules for Linear Integer Arithmetic, and show how they are scheduled with the other rules of Spacer in an efficient verification algorithm. The new global rules are summarized in Algorithm 2. We use the same guarded command notation as in description of Spacer in Algorithm 1. Note that the rules supplement, and not replace, the ones in Algorithm 1.

*Subsume* is the most natural rule to explain. It says that if there is a set of lemmas $\mathcal {L}$ at level *i*, and there exists a formula $\psi$ such that (a) $\psi$ is stronger than every lemma in $\mathcal {L}$, and (b) $\psi$ over-approximates states reachable in at most *k* steps, where $k \ge i$, then $\psi$ can be added to the trace to subsume $\mathcal {L}$. This rule reduces the size of the global proof – that is, the number of total not-subsumed lemmas. Note that the rule allows $\psi$ to be at a level *k* that is higher than *i*. The choice of $\psi$ is left open. The details are likely to be specific to the theory involved. For example, when instantiated for LIA, Subsume is sufficient to solve example in Fig. 1(a). Interestingly, Subsume is not likely to be effective for propositional IC3. In that case, $\psi$ is a clause and the only way for it to be stronger than $\mathcal {L}$ is for $\psi$ to be a syntactic sub-sequence of every lemma in $\mathcal {L}$, but such $\psi$ is already explored by local inductive generalization (rule Induction in Algorithm 1).

*Concretize* applies to a pob, unlike Subsume. It is motivated by example in Fig. 1(b) that highlights the problem of excessive local generalization. Spacer always computes as general predecessors as possible. This is necessary for refutational completeness since in an infinite state system there are infinitely many potential predecessors. Computing the most general predecessor ensures that Spacer finds a counterexample, if it exists. However, this also forces Spacer to discover more general, and sometimes more complex, lemmas than might be necessary for an inductive invariant. Without a global view of the overall proof, it is hard to determine when the algorithm generalizes too much. The intuition for Concretize is that generalization is excessive when there is a single pob $\langle \varphi , j \rangle$ that is not blocked, yet, there is a set of lemmas $\mathcal {L}$ such that every lemma $\ell \in \mathcal {L}$ partially blocks $\varphi$. That is, for any $\ell \in \mathcal {L}$, there is a sub-region $\varphi _{\ell }$ of pob $\varphi$ that is blocked by $\ell$ (i.e., $\ell \Rightarrow \lnot \varphi _{\ell }$), and there is at least one state $s \in \varphi$ that is not blocked by any existing lemma in $\mathcal {L}$ (i.e., $s \models \varphi \wedge \bigwedge \mathcal {L}$). In this case, Concretize computes an under-approximation $\gamma$ of $\varphi$ that includes some not-yet-blocked state *s*. The new pob is added to the lowest level at which $\gamma$ is not yet blocked. Concretize is useful to solve the example in Fig. 1(b).

*Conjecture* guides the algorithm away from being stuck in the same part of the search space. A single pob $\varphi$ might be blocked by a different lemma at each level that $\varphi$ appears in. This indicates that the lemmas are too strong, and cannot be propagated successfully to a higher level. The goal of the Conjecture rule is to identify such a case to guide the algorithm to explore alternative proofs with a better potential for generalization. This is done by abstracting away the part of the pob that has been blocked in the past. The pre-condition for Conjecture is the existence of a pob $\langle \varphi , j \rangle$ such that $\varphi$ is split into two (not necessarily disjoint) sets of literals, $\alpha$ and $\beta$. Second, there must be a set of lemmas $\mathcal {L}$, at a (typically much lower) level $i < j$ such that every lemma $\ell \in \mathcal {L}$ blocks $\varphi$, and, moreover, blocks $\varphi$ by blocking $\beta$. Intuitively, this implies that while there are many different lemmas (i.e., all lemmas in $\mathcal {L}$) that block $\varphi$ at different levels, all of them correspond to a *local* generalization of $\lnot \beta$ that could not be propagated to block $\varphi$ at higher levels. In this case, Conjecture abstracts the pob $\varphi$ into $\alpha$, hoping to generate an alternative way to block $\varphi$. Of course, $\alpha$ is conjectured only if it is not already blocked and does not contain any known reachable states. Conjecture is necessary for a quick convergence on the example in Fig. 1(c). In some respect, Conjecture is akin to widening in Abstract Interpretation [12] – it abstracts a set of states by dropping constraints that appear to prevent further exploration. Of course, it is also quite different since it does not guarantee termination. While Conjecture is applicable to propositional IC3 as well, it is much more significant in SMT-based setting since in many FOL theories a single literal in a pob might result in infinitely many distinct lemmas.

Each of the rules can be applied by itself, but they are most effective in combination. For example, Concretize creates less general predecessors, that, in the worst case, lead to many simple lemmas. At the same time, Subsume combines lemmas together into more complex ones. The interaction of the two produces lemmas that neither one can produce in isolation. At the same time, Conjecture helps unstuck the algorithm from a single unproductive pob, allowing the other rules to take effect.

## Global Guidance for Linear Integer Arithmetic

In this section, we present a specialization of our general rules, shown in Algorithm 2, to the theory of Linear Integer Arithmetic (LIA). This requires solving two problems: identifying subsets of lemmas for pre-conditions of the rules (clearly using all possible subsets is too expensive), and applying the rule once its pre-condition is met. For lemma selection, we introduce a notion of syntactic clustering based on anti-unification. For rule application, we exploit basic properties of LIA for an effective algorithm. Our presentation is focused on LIA exclusively. However, the rules extend to combinations of LIA with other theories, such as the combined theory of LIA and Arrays.

The rest of this section is structured as follows. We begin with a brief background on LIA in Sect. 4.1. We then present our lemma selection scheme, which is common to all the rules, in Sect. 4.2, followed by a description of how the rules Subsume (in Sect. 4.3), Concretize (in Sect. 4.4), and Conjecture (in Sect. 4.5) are instantiated for LIA. We conclude in Sect. 4.6 with an algorithm that integrates all the rules together.

### Linear Integer Arithmetic: Background

In the theory of Linear Integer Arithmetic (LIA), formulas are defined over a signature that includes interpreted function symbols $+$, −, $\times$, interpreted predicate symbols <, $\le$, $\mid$, interpreted constant symbols $0,1,2,\ldots$, and uninterpreted constant symbols $a, b,\ldots , x,y,\ldots$. We write $\mathbb {Z}$ for the set interpreted constant symbols, and call them *integers*. We use *constants* to refer exclusively to the uninterpreted constants (these are often called *variables* in LIA literature). Terms (and accordingly formulas) in LIA are restricted to be *linear*, that is, multiplication is never applied to two constants.

We write $\textsc {LIA}^{-\text {div}}$ for the fragment of LIA that excludes divisiblity (d$\mid$h) predicates. A literal in $\textsc {LIA}^{-\text {div}}$ is a linear inequality; a cube is a conjunction of such inequalities, that is, a polytope. We find it convenient to use matrix-based notation for representing cubes in $\textsc {LIA}^{-\text {div}}$. A ground cube $c \in \textsc {LIA}^{-\text {div}}$ with *p* inequalities (literals) over *k* (uninterpreted) constants is written as $A \cdot \textit{\textbf{x}}\le \textit{\textbf{n}}$, where *A* is a $p \times k$ matrix of coefficients in $\mathbb {Z}^{p \times k}$, $\textit{\textbf{x}}= (x_1 \cdots x_k)^T$ is a column vector that consists of the (uninterpreted) constants, and $\textit{\textbf{n}}= ( n_1 \cdots n_p)^T$ is a column vector in $\mathbb {Z}^p$. For example, the cube $x \ge 2 \wedge 2x + y \le 3$ is written as  In the sequel, all vectors are column vectors, super-script *T* denotes transpose, dot is used for a dot product and $[\textit{\textbf{n}}_1 ; \textit{\textbf{n}}_2]$ stands for a matrix of column vectors $\textit{\textbf{n}}_1$ and $\textit{\textbf{n}}_2$.

### Lemma Selection

A common pre-condition for all of our global rules in Algorithm 2 is the existence of a subset of lemmas $\mathcal {L}$ of some frame $\mathcal {O}_i$. Attempting to apply the rules for every subset of $\mathcal {O}_i$ is infeasible. In practice, we use syntactic similarity between lemmas as a predictor that one of the global rules is applicable, and restrict $\mathcal {L}$ to subsets of syntactically similar lemmas. In the rest of this section, we formally define what we mean by *syntactic similarity*, and how syntactically similar subsets of lemmas, called *clusters*, are maintained efficiently throughout the algorithm.

*Syntactic Similarity.* A formula $\pi$ with free variables is called a *pattern*. Note that we do not require $\pi$ to be in LIA. Let $\sigma$ be a substitution, i.e., a mapping from variables to terms. We write $\pi \sigma$ for the result of replacing all occurrences of free variables in $\pi$ with their mapping under $\sigma$. A substitution $\sigma$ is called *numeric* if it maps every variable to an integer, i.e., the range of $\sigma$ is $\mathbb {Z}$. We say that a formula $\varphi$ *numerically matches* a pattern $\pi$ iff there exists a numeric substitution $\sigma$ such that $\varphi = \pi \sigma$. Note that, as usual, the equality is syntactic. For example, consider the pattern $\pi = v_0a + v_1b \le 0$ with free variables $v_0$ and $v_1$ and uninterpreted constants *a* and *b*. The formula $\varphi _1 = 3a + 4b \le 0$ matches $\pi$ via a numeric substitution $\sigma _1 = \{v_0 \mapsto 3, v_1 \mapsto 4\}$. However, $\varphi _2 = 4b + 3a \le 0$, while semantically equivalent to $\varphi _1$, does not match $\pi$. Similarly $\varphi _3 = a + b \le 0$ does not match $\pi$ as well.

Matching is extended to patterns in the usual way by allowing a substitution $\sigma$ to map variables to variables. We say that a pattern $\pi _1$ is more general than a pattern $\pi _2$ if $\pi _2$ matches $\pi _1$. A pattern $\pi$ is a *numeric anti-unifier* for a pair of formulas $\varphi _1$ and $\varphi _2$ if both $\varphi _1$ and $\varphi _2$ match $\pi$ numerically. We write $\textit{anti}(\varphi _1, \varphi _2)$ for a most general numeric anti-unifier of $\varphi _1$ and $\varphi _2$. We say that two formulas $\varphi _1$ and $\varphi _2$ are *syntactically similar* if there exists a numeric anti-unifier between them (i.e., $\textit{anti}(\varphi _1, \varphi _2)$ is defined). Anti-unification is extended to sets of formulas in the usual way.

*Clusters.* We use anti-unification to define *clusters* of syntactically similar formulas. Let $\varPhi$ be a fixed set of formulas, and $\pi$ a pattern. A *cluster*, $\mathcal {C}_{\varPhi }(\pi )$, is a subset of $\varPhi$ such that every formula $\varphi \in \mathcal {C}_{\varPhi }(\pi )$ numerically matches $\pi$. That is, $\pi$ is a numeric anti-unifier for $\mathcal {C}_{\varPhi }(\pi )$. In the implementation, we restrict the pre-conditions of the global rules so that a subset of lemmas $\mathcal {L}\subseteq \mathcal {O}_i$ is a cluster for some pattern $\pi$, i.e., $\mathcal {L}= \mathcal {C}_{\mathcal {O}_i}(\pi )$.

*Clustering Lemmas.* We use the following strategy to efficiently keep track of available clusters. Let $\ell _{\text {new}}$ be a new lemma to be added to $\mathcal {O}_i$. Assume there is at least one lemma $\ell \in \mathcal {O}_i$ that numerically anti-unifies with $\ell _{\text {new}}$ via some pattern $\pi$. If such an $\ell$ does not belong to any cluster, a new cluster $\mathcal {C}_{\mathcal {O}_i}(\pi ) = \{\ell _{\text {new}}, \ell \}$ is formed, where $\pi = \textit{anti}(\ell _{\text {new}}, \ell )$. Otherwise, for every lemma $\ell \in \mathcal {O}_{i}$ that numerically matches $\ell _{\text {new}}$ and every cluster $\mathcal {C}_{\mathcal {O}_i}(\hat{\pi })$ containing $\ell$, $\ell _{\text {new}}$ is added to $\mathcal {C}_{\mathcal {O}_i}(\hat{\pi })$ if $\ell _{\text {new}}$ matches $\hat{\pi }$, or a new cluster is formed using $\ell$, $\ell _{\text {new}}$, and any other lemmas in $\mathcal {C}_{\mathcal {O}_i}(\hat{\pi })$ that anti-unify with them. Note that a new lemma $\ell _{\text {new}}$ might belong to multiple clusters.

For example, suppose $\ell _{\text {new}}= (a \le 6 \vee b \le 6)$, and there is already a cluster $\mathcal {C}_{\mathcal {O}_i}(a \le v_0 \vee b \le 5) = \{ (a \le 5 \vee b \le 5), (a \le 8 \vee b \le 5)\}$. Since $\ell _{\text {new}}$ anti-unifies with each of the lemmas in the cluster, but does not match the pattern $a \le v_0 \vee b \le 5$, a new cluster that includes all of them is formed w.r.t. a more general pattern: $\mathcal {C}_{\mathcal {O}_i}(a \le v_0 \vee b \le v_1) = \{ (a \le 6 \vee b \le 6), (a \le 5 \vee b \le 5), (a \le 8 \vee b \le 5)\}$.

In the presentation above, we assumed that anti-unification is completely syntactic. This is problematic in practice since it significantly limits the applicability of the global rules. Recall, for example, that $a + b \le 0$ and $2a + 2b \le 0$ do not anti-unify numerically according to our definitions, and, therefore, do not cluster together. In practice, we augment syntactic anti-unification with simple rewrite rules that are applied greedily. For example, we normalize all $\textsc {LIA}$ terms, take care of implicit multiplication by 1, and of associativity and commutativity of addition. In the future, it is interesting to explore how advanced anti-unification algorithms, such as [8, 27], can be adapted for our purpose.

### Subsume Rule for LIA

Recall that the Subsume rule (Algorithm 2) takes a cluster of lemmas $\mathcal {L}= \mathcal {C}_{\mathcal {O}_i}(\pi )$ and computes a new lemma $\psi$ that subsumes all the lemmas in $\mathcal {L}$, that is $\psi \Rightarrow \bigwedge \mathcal {L}$. We find it convenient to dualize the problem. Let $\mathcal {S}= \{ \lnot \ell \mid \ell \in \mathcal {L}\}$ be the dual of $\mathcal {L}$, clearly $\psi \Rightarrow \bigwedge \mathcal {L}$ iff $(\bigvee \mathcal {S}) \Rightarrow \lnot \psi$. Note that $\mathcal {L}$ is a set of clauses, $\mathcal {S}$ is a set of cubes, $\psi$ is a clause, and $\lnot \psi$ is a cube. In the case of $\textsc {LIA}^{-\text {div}}$, this means that $\bigvee \mathcal {S}$ represents a union of convex sets, and $\lnot \psi$ represents a convex set that the Subsume rule must find. The strongest such $\lnot \psi$ in $\textsc {LIA}^{-\text {div}}$ exists, and is the convex closure of $\mathcal {S}$. Thus, applying Subsume in the context of $\textsc {LIA}^{-\text {div}}$ is reduced to computing a convex closure of a set of (negated) lemmas in a cluster. Full LIA extends $\textsc {LIA}^{-\text {div}}$ with divisibility constraints. Therefore, Subsume obtains a stronger $\lnot \psi$ by adding such constraints.

#### Example 1

For example, consider the following cluster:

$$\begin{aligned} \mathcal {L}&= \{ (x> 2 \vee x< 2 \vee y> 3), (x> 4 \vee x< 4 \vee y> 5), (x> 8 \vee x < 8 \vee y > 9) \} \\ \mathcal {S}&= \{(x \le 2 \wedge x \ge 2 \wedge y \le 3), (x \ge 4 \wedge x \le 4 \wedge y \le 5), (x \ge 8 \wedge x \le 8 \wedge y \le 9) \} \end{aligned}$$

The convex closure of $\mathcal {S}$ in $\textsc {LIA}^{-\text {div}}$ is $2 \le x \le 8 \wedge y \le x + 1$. However, a stronger over-approximation exists in LIA: $2 \le x \le 8 \wedge y \le x + 1 \wedge (2 \mid x)$.    $\square$

In the sequel, we describe subsumeCube (Algorithm 3) which computes a cube $\varphi$ that over-approximates $(\bigvee \mathcal {S})$. Subsume is then implemented by removing from $\mathcal {L}$ lemmas that are already subsumed by existing lemmas in $\mathcal {L}$, dualizing the result into $\mathcal {S}$, invoking subsumeCube on $\mathcal {S}$ and returning $\lnot \varphi$ as a lemma that subsumes $\mathcal {L}$.

Recall that Subsume is tried only in the case $\mathcal {L}= \mathcal {C}_{\mathcal {O}_i}(\pi )$. We further require that the negated pattern, $\lnot \pi$, is of the form $A \cdot \textit{\textbf{x}}\le \textit{\textbf{v}}$, where *A* is a coefficients matrix, $\textit{\textbf{x}}$ is a vector of constants and $\textit{\textbf{v}}= (v_1 \cdots v_p)^T$ is a vector of *p* free variables. Under this assumption, $\mathcal {S}$ (the dual of $\mathcal {L}$) is of the form $\{ (A \cdot \textit{\textbf{x}}\le \textit{\textbf{n}}_i) \mid 1 \le i \le q\}$, where $q = |\mathcal {S}|$, and for each $1 \le i \le q$, $\textit{\textbf{n}}_i$ is a numeric substitution to $\textit{\textbf{v}}$ from which one of the negated lemmas in $\mathcal {S}$ is obtained. That is, $|\textit{\textbf{n}}_i| = |\textit{\textbf{v}}|$. In Example 1, $\lnot \pi = x \le v_1 \wedge -x \le v_2 \wedge y \le v_3$ andEach cube $(A \cdot \textit{\textbf{x}}\le \textit{\textbf{n}}_i )\in \mathcal {S}$ is equivalent to . Finally, . Thus, computing the over-approximation of $\mathcal {S}$ is reduced to (a) computing the convex hull *H* of a set of points $\{\textit{\textbf{n}}_i \mid 1 \le i \le q\}$, (b) computing divisibility constraints *D* that are satisfied by all the points, (c) substituting $H \wedge D$ for the disjunction in the equation above, and (c) eliminating variables $\textit{\textbf{v}}$. Both the computation of $H \wedge D$ and the elimination of $\textit{\textbf{v}}$ may be prohibitively expensive. We, therefore, over-approximate them. Our approach for doing so is presented in Algorithm 3, and explained in detail below.

*Computing the convex hull of *$\{\textit{\textbf{n}}_i \mid 1 \le i \le q\}$. lines 3 to 8 compute the convex hull of $\{\textit{\textbf{n}}_i \mid 1 \le i \le q\}$ as a formula over $\textit{\textbf{v}}$, where variable $v_j$, for $1\le j \le p$, represents the $j^\text {th}$ coordinates in the vectors (points) $\textit{\textbf{n}}_i$. Some of the coordinates, $v_j$, in these vectors may be linearly dependent upon others. To simplify the problem, we first identify such dependencies and compute a set of linear equalities that expresses them (*L* in line 4). To do so, we consider a matrix $N_{q \times p}$, where the $i^\text {th}$ row consists of $\textit{\textbf{n}}_i^T$. The $j^\text {th}$ column in *N*, denoted $N_{*j}$, corresponds to the $j^\text {th}$ coordinate, $v_j$. The rank of *N* is the number of linearly independent columns (and rows). The other columns (coordinates) can be expressed by linear combinations of the linearly independent ones. To compute these linear combinations we use the kernel of $[N ; \textit{\textbf{1}}]$ (*N* appended with a column vector of 1’s), which is the set of all vectors $\textit{\textbf{y}}$ such that $[N ; \textit{\textbf{1}}]\cdot \textit{\textbf{y}} = \textit{\textbf{0}}$, where $\textit{\textbf{0}}$ is the zero vector. Let $B = \text {kernel}([N ; \textit{\textbf{1}}])$ be a basis for the kernel of $[N ; \textit{\textbf{1}}]$. Then $|B| = p - \text {rank}(N)$, and for each vector $\textit{\textbf{y}} \in B$, the linear equality $[ v_1 \cdots v_p \; 1] \cdot \textit{\textbf{y}} = 0$ holds in all the rows of *N* (i.e., all the given vectors satisfy it). We accumulate these equalities, which capture the linear dependencies between the coordinates, in *L*. Further, the equalities are used to compute $\text {rank}(N)$ coordinates (columns in *N*) that are linearly independent and, modulo *L*, uniquely determine the remaining coordinates. We denote by $\textit{\textbf{v}}^{L_{\downarrow }}$ the subset of $\textit{\textbf{v}}$ that consists of the linearly independent coordinates. We further denote by $\textit{\textbf{n}}_i^{L_{\downarrow }}$ the projection of $\textit{\textbf{n}}_i$ to these coordinates and by $N^{L_{\downarrow }}$ the projection of *N* to the corresponding columns. We have that $(\bigvee (\textit{\textbf{v}}= \textit{\textbf{n}}_i)) \equiv L \wedge (\bigvee (\textit{\textbf{v}}^{L_{\downarrow }} = \textit{\textbf{n}}_i^{L_{\downarrow }})$.

In Example 1, the numeral matrix is , for which . Therefore, *L* is the conjunction of equalities $v_1 + v_2 = 0 \wedge v_1 - v_3 + 1 = 0$, or, equivalently $v_3 = v_1 + 1 \wedge v_2 = -v_1$, $$\textit{\textbf{v}}^{L_{\downarrow }} = \begin{pmatrix} v_1 \end{pmatrix}^T$$, and

$$\begin{aligned} \textit{\textbf{n}}_1^{L_{\downarrow }} = \begin{bmatrix} 2 \end{bmatrix} \qquad \textit{\textbf{n}}_2^{L_{\downarrow }} = \begin{bmatrix} 4 \end{bmatrix} \qquad \textit{\textbf{n}}_3^{L_{\downarrow }} = \begin{bmatrix} 8 \end{bmatrix} \qquad N^{L_{\downarrow }} = \begin{bmatrix} 2 \\ 4 \\ 8 \end{bmatrix} \end{aligned}$$

Next, we compute the convex closure of $\bigvee (\textit{\textbf{v}}^{L_{\downarrow }} = \textit{\textbf{n}}_i^{L_{\downarrow }})$, and conjoin it with *L* to obtain *H*, the convex closure of $(\bigvee (\textit{\textbf{v}}= \textit{\textbf{n}}_i))$.

If the dimension of $\textit{\textbf{v}}^{L_{\downarrow }}$ is one, as is the case in the example above, convex closure, *C*, of $\bigvee (\textit{\textbf{v}}^{L_{\downarrow }} = \textit{\textbf{n}}_i^{L_{\downarrow }})$ is obtained by bounding the sole element of $\textit{\textbf{v}}^{L_{\downarrow }}$ based on its values in $N^{L_{\downarrow }}$ (line 6). In Example 1, we obtain $C = 2 \le v_1 \le 8$.

If the dimension of $\textit{\textbf{v}}^{L_{\downarrow }}$ is greater than one, just computing the bounds of one of the constants is not sufficient. Instead, we use the concept of syntactic convex closure from [2] to compute the convex closure of $\bigvee (\textit{\textbf{v}}^{L_{\downarrow }} = \textit{\textbf{n}}_i^{L_{\downarrow }})$ as  where $\varvec{\alpha }$ is a vector that consists of *q* fresh *rational* variables and *C* is defined as follows (line 8): $C = \varvec{\alpha }\ge 0 \wedge \varSigma \varvec{\alpha }= 1 \wedge \varvec{\alpha }^T \cdot N^{L_{\downarrow }} = (\textit{\textbf{v}}^{L_{\downarrow }})^T$. *C* states that $(\textit{\textbf{v}}^{L_{\downarrow }})^T$ is a convex combination of the rows of $N^{L_{\downarrow }}$, or, in other words, $\textit{\textbf{v}}^{L_{\downarrow }}$ is a convex combination of $\{ \textit{\textbf{n}}_i^{L_{\downarrow }} \mid 1 \le i \le q\}$.

To illustrate the syntactic convex closure, consider a second example with a set of cubes: $$ \mathcal {S}= \{(x \le 0 \wedge y \le 6), (x \le 6 \wedge y \le 0), (x \le 5 \wedge y \le 5)\}$$. The coefficient matrix *A*, and the numeral matrix *N* are then:  and . Here, $$\text {kernel}([N ; \textit{\textbf{1}}])$$ is empty – all the columns are linearly independent, hence, $$L = true $$ and $$\textit{\textbf{v}}^{L_{\downarrow }} = \textit{\textbf{v}}$$. Therefore, syntactic convex closure is applied to the full matrix *N*, resulting in

$$\begin{aligned}&C = (\alpha _1 \ge 0) \wedge (\alpha _2 \ge 0) \wedge (\alpha _3 \ge 0) \wedge (\alpha _1 + \alpha _2 + \alpha _3 = 1) \wedge {} \\&\qquad \qquad \qquad \qquad \qquad \qquad \qquad \qquad \,\,\; (6\alpha _2 + 5\alpha _3 = v_1) \wedge (6\alpha _1 + 5\alpha _3 = v_2) \end{aligned}$$

The convex closure of $\bigvee ({\textit{\textbf{v}}} = {\textit{\textbf{n}}_i})$ is then , which is  here.

*Divisibility Constraints.* Inductive invariants for verification problems often require divisibility constraints. We, therefore, use such constraints, denoted *D*, to obtain a stronger over-approximation of $\bigvee ({\textit{\textbf{v}}} = {\textit{\textbf{n}}_i})$ than the convex closure. To add a divisibility constraint for $v_j \in \textit{\textbf{v}}^{L_{\downarrow }}$, we consider the column $N_{*j}^{L_{\downarrow }}$ that corresponds to $v_j$ in $N^{L_{\downarrow }}$. We find the largest positive integer *d* such that each integer in $N_{*j}^{L_{\downarrow }}$ leaves the same remainder when divided by *d*; namely, there exists $0 \le r < d$ such that $n \bmod d = r$ for every $n \in N_{*j}^{L_{\downarrow }}$. This means that $d \mid (v_j - r)$ is satisfied by all the points $\textit{\textbf{n}}_i$. Note that such *r* always exists for $d=1$. To avoid this trivial case, we add the constraint $d \mid (v_j - r)$ only if $d \ne 1$ (line 12). We repeat this process for each $v_j \in \textit{\textbf{v}}^{L_{\downarrow }}$.

In Example 1, all the elements in the (only) column of the matrix $N^{L_{\downarrow }}$, which corresponds to $v_1$, are divisible by 2, and no larger *d* has a corresponding *r*. Thus, line 12 of Algorithm 3 adds the divisibility condition $(2 \mid v_1)$ to *D*.

*Eliminating Existentially Quantified Variables Using MBP.* By combining the linear equalities exhibited by *N*, the convex closure of $N^{L_{\downarrow }}$ and the divisibility constraints on $\textit{\textbf{v}}$, we obtain  as an over-approximation of $\bigvee ({\textit{\textbf{v}}} = {\textit{\textbf{n}}_i})$. Accordingly, , where $\psi = (A \cdot \textit{\textbf{x}}\le \textit{\textbf{v}}) \wedge L \wedge C \wedge D$, is an over-approximation of  (line 13). In order to get a LIA cube that overapproximates $\bigvee \mathcal {S}$, it remains to eliminate the existential quantifiers. Since quantifier elimination is expensive, and does not necessarily generate convex formulas (cubes), we approximate it using MBP. Namely, we obtain a cube $\varphi$ that under-approximates  by applying MBP on $\psi$ and a model $M_0 \models \psi$. We then use an SMT solver to drop literals from $\varphi$ until it over-approximates , and hence also $\bigvee \mathcal {S}$ (lines 16 to 19). The result is returned by Subsume as an over-approximation of $\bigvee \mathcal {S}$.

Models $M_0$ that satisfy $\psi$ and do not satisfy any of the cubes in $\mathcal {S}$ are preferred when computing MBP (line 14) as they ensure that the result of MBP is not subsumed by any of the cubes in $\mathcal {S}$.

Note that the $\varvec{\alpha }$ are rational variables and $\textit{\textbf{v}}$ are integer variables, which means we require MBP to support a mixture of integer and rational variables. To achieve this, we first relax all constants to be rationals and apply MBP over LRA to eliminate $\varvec{\alpha }$. We then adjust the resulting formula back to integer arithmetic by multiplying each atom by the least common multiple of the denominators of the coefficients in it. Finally, we apply MBP over the integers to eliminate $\textit{\textbf{v}}$.

Considering Example 1 again, we get that $\psi = (x \le v_1) \wedge (-x \le v_2) \wedge (y \le v_3) \wedge (v_3 = 1 + v_1) \wedge (v_2 = -v_1) \wedge (2 \le v_1 \le 8) \wedge (2 \mid v_1)$ (the first three conjuncts correspond to $(A \cdot (x\; y)^T \le (v_1\; v_2 \; v_3)^T)$). Note that in this case we do not have rational variables $\varvec{\alpha }$ since $|\textit{\textbf{v}}^{L_{\downarrow }}| = 1$. Depending on the model, the result of MBP can be one of

$$\begin{aligned} y \le x + 1 \wedge 2 \le x \le 8 \wedge (2 \mid y - 1) \wedge (2 \mid x)&\qquad x \ge 2 \wedge x \le 2 \wedge y \le 3&\\ y \le x + 1 \wedge 2 \le x \le 8 \wedge (2 \mid x)&\qquad x \ge 8 \wedge x \le 8 \wedge y \le 9&\\ y \ge x + 1 \wedge y \le x + 1 \wedge 3 \le y \le 9 \wedge (2 \mid y - 1)&\qquad \end{aligned}$$

However, we prefer a model that does not satisfy any cube in $\mathcal {S}= \{(x \ge 2 \wedge x \le 2 \wedge y \le 3), (x \le 4 \wedge x \ge 4 \wedge y \le 5), (x \le 8 \wedge x \ge 8 \wedge y \le 9) \}$, rules off the two possibilities on the right. None of these cubes cover $\psi$, hence generalization is used.

If the first cube is obtained by MBP, it is generalized into $y \le x + 1 \wedge x \ge 2 \wedge x \le 8 \wedge (2{\mid }x)$; the second cube is already an over-approximation; the third cube is generalized into $y \le x + 1 \wedge y \le 9$. Indeed, each of these cubes over-approximates $\bigvee \mathcal {S}$.
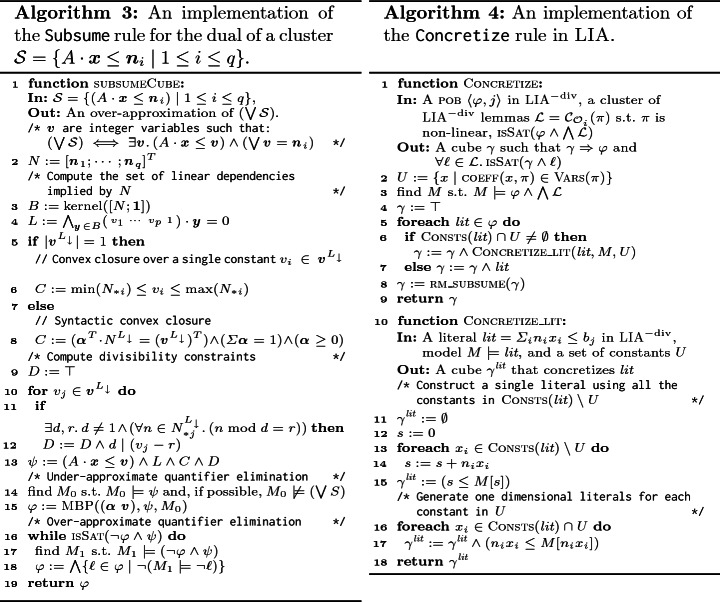


### Concretize Rule for LIA

The Concretize rule (Algorithm 2) takes a cluster of lemmas $\mathcal {L}= \mathcal {C}_{\mathcal {O}_i}(\pi )$ and a pob $\langle \varphi , j \rangle$ such that each lemma in $\mathcal {L}$ partially blocks $\varphi$, and creates a new pob $\gamma$ that is still not blocked by $\mathcal {L}$, but $\gamma$ is more concrete, i.e., $\gamma \Rightarrow \varphi$. In our implementation, this rule is applied when $\varphi$ is in $\textsc {LIA}^{-\text {div}}$. We further require that the pattern, $\pi$, of $\mathcal {L}$ is non-linear, i.e., some of the constants appear in $\pi$ with free variables as their coefficients. We denote these constants by *U*. An example is the pattern $\pi = v_0 x + v_1 y + z \le 0$, where $U = \{x,y\}$. Having such a cluster is an indication that attempting to block $\varphi$ in full with a single lemma may require to track non-linear correlations between the constants, which is impossible to do in LIA. In such cases, we identify the coupling of the constants in *U* in pobs (and hence in lemmas) as the potential source of non-linearity. Hence, we concretize (strengthen) $\varphi$ into a pob $\gamma$ where the constants in *U* are no longer coupled to any other constant.

*Coupling.* Formally, constants *u* and *v* are *coupled* in a cube *c*, denoted $u \bowtie _{c} v$, if there exists a literal $\textit{lit}$ in *c* such that both *u* and *v* appear in $\textit{lit}$ (i.e., their coefficients in $\textit{lit}$ are non-zero). For example, *x* and *y* are coupled in $x + y \le 0 \wedge z \le 0$ whereas neither of them are coupled with *z*. A constant *u* is said to be *isolated* in a cube *c*, denoted $\textsc {Iso}(u, c)$, if it appears in *c* but it is not coupled with any other constant in *c*. In the above cube, *z* is isolated.

*Concretization by Decoupling.* Given a pob $\varphi$ (a cube) and a cluster $\mathcal {L}$, Algorithm 4 presents our approach for concretizing $\varphi$ by decoupling the constants in *U*—those that have variables as coefficients in the pattern of $\mathcal {L}$ (line 2). Concretization is guided by a model $M \models \varphi \wedge \bigwedge \mathcal {L}$, representing a part of $\varphi$ that is not yet blocked by the lemmas in $\mathcal {L}$ (line 3). Given such *M*, we concretize $\varphi$ into a *model-preserving* under-approximation that isolates all the constants in *U* and preserves all other couplings. That is, we find a cube $\gamma$, such that1Note that $\gamma$ is not blocked by $\mathcal {L}$ since *M* satisfies both $\bigwedge \mathcal {L}$ and $\gamma$. For example, if $\varphi = (x + y \le 0) \wedge (x - y \le 0) \wedge (x + z \ge 0)$ and $M = [x = 0, y = 0, z = 1]$, then $\gamma = 0 \le y \le 0 \wedge x \le 0 \wedge x + z \ge 1$ is a model preserving under-approximation that isolates $U = \{y\}$.

Algorithm 4 computes such a cube $\gamma$ by a point-wise concretization of the literals of $\varphi$ followed by the removal of subsumed literals. Literals that do not contain constants from *U* remain unchanged. A literal of the form $\textit{lit}= t \le b$, where $t=\sum _i n_ix_i$ (recall that every literal in $\textsc {LIA}^{-\text {div}}$ can be normalized to this form), that includes constants from *U* is concretized into a *cube* by (1) isolating each of the summands $n_i x_i$ in *t* that include *U* from the rest, and (2) for each of the resulting sub-expressions creating a literal that uses its value in *M* as a bound. Formally, *t* is decomposed to $s + \sum _{x_i \in U} n_i x_i$, where $s = \sum _{x_i \not \in U} n_i x_i$. The concretization of $\textit{lit}$ is the cube $\gamma ^{\textit{lit}} = s \le M[s] \wedge \bigwedge _{x_i \in U} n_i x_i \le M[n_i x_i]$, where $M[t']$ denotes the interpretation of $t'$ in *M*. Note that $\gamma ^{\textit{lit}} \Rightarrow \textit{lit}$ since the bounds are stronger than the original bound on *t*: $M[s] + \sum _{x_i \in U} M[n_i x_i] = M[t] \le b$. This ensures that $\gamma$, obtained by the conjunction of literal concretizations, implies $\varphi$. It trivially satisfies the other conditions of Eq. (1).

For example, the concretization of the literal $(x + y \le 0)$ with respect to $U = \{y\}$ and $M = [x = 0, y = 0, z = 1]$ is the cube $x \le 0 \wedge y \le 0$. Applying concretization in a similar manner to all the literals of the cube $\varphi = (x + y \le 0) \wedge (x - y \le 0) \wedge (x + z \ge 0)$ from the previous example, we obtain the concretization $x \le 0 \wedge 0 \le y \le 0 \wedge x + z \ge 0$. Note that the last literal is not concretized as it does not include *y*.

### Conjecture Rule for LIA

The Conjecture rule (see Algorithm 2) takes a set of lemmas $\mathcal {L}$ and a pob $\varphi \equiv \alpha \wedge \beta$ such that all lemmas in $\mathcal {L}$ block $\beta$, but none of them blocks $\alpha$, where $\alpha$ does not include any known reachable states. It returns $\alpha$ as a new pob.

For LIA, Conjecture is applied when the following conditions are met: (1) the pob $\varphi$ is of the form $\varphi _1 \wedge \varphi _2 \wedge \varphi _3$, where $\varphi _3 = (\textit{\textbf{n}}^T \cdot \textit{\textbf{x}}\le b)$, and $\varphi _1$ and $\varphi _2$ are any cubes. The sub-cube $\varphi _1 \wedge \varphi _2$ acts as $\alpha$, while the sub-cube $\varphi _2 \wedge \varphi _3$ acts as $\beta$. (2) The cluster $\mathcal {L}$ consists of $\{bg \vee (\textit{\textbf{n}}^T \cdot \textit{\textbf{x}}\ge b_i) \mid 1 \le i \le q\}$, where $b_i > b$ and $bg \Rightarrow \lnot \varphi _2$. This means that each of the lemmas in $\mathcal {L}$ blocks $\beta = \varphi _2 \wedge \varphi _3$, and they may be ordered as a sequence of increasingly stronger lemmas, indicating that they were created by trying to block the pob at different levels, leading to too strong lemmas that failed to propagate to higher levels. (3) The formula $(bg \vee (\textit{\textbf{n}}^T \cdot \textit{\textbf{x}}\ge b_i)) \wedge \varphi _1 \wedge \varphi _2$ is satisfiable, that is, none of the lemmas in $\mathcal {L}$ block $\alpha =\varphi _1 \wedge \varphi _2$, and (4) $\mathcal {U}\Rightarrow \lnot (\varphi _1 \wedge \varphi _2)$, that is, no state in $\varphi _1 \wedge \varphi _2$ is known to be reachable. If all four conditions are met, we conjecture $\alpha = \varphi _1 \wedge \varphi _2$. This is implemented by conjecture, that returns $\alpha$ (or $\bot$ when the pre-conditions are not met).

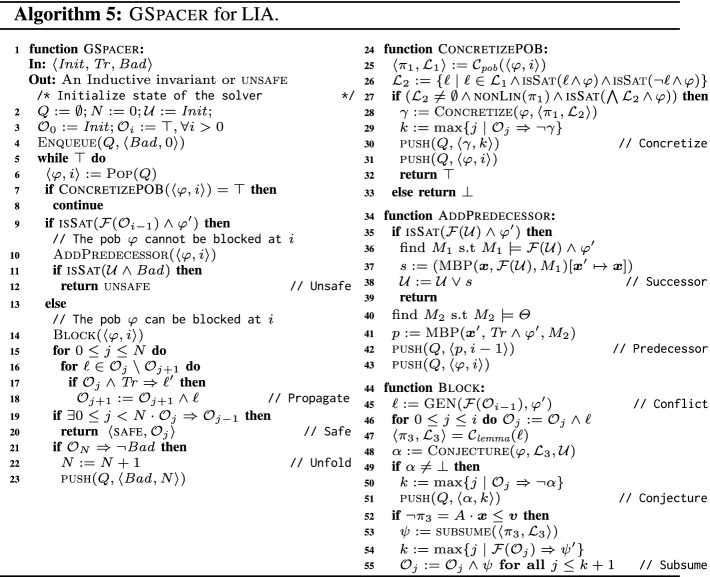



For example, consider the pob $\varphi = x \ge 10 \wedge (x + y \ge 10) \wedge y \le 10$ and a cluster of lemmas $\mathcal {L}= \{ (x + y \le 0 \vee y \ge 101), (x + y \le 0 \vee y \ge 102)\}$. In this case, $\varphi _1 = x \ge 10$, $\varphi _2 = (x + y \ge 10)$, $\varphi _3 = y \le 10$, and $bg =x + y \le 0$. Each of the lemmas in $\mathcal {L}$ block $\varphi _2 \wedge \varphi _3$ but none of them block $\varphi _1 \wedge \varphi _2$. Therefore, we conjecture $\varphi _1 \wedge \varphi _2$: $x \ge 10 \wedge (x + y \ge 10)$.

### Putting It All Together

Having explained the implementation of the new rules for LIA, we now put all the ingredients together into an algorithm, GSpacer. In particular, we present our choices as to when to apply the new rules, and on which clusters of lemmas and pobs. As can be seen in Sect. 5, this implementation works very well on a wide range of benchmarks.

Algorithm 5 presents GSpacer. The comments to the right side of a line refer to the abstract rules in Algorithm 1 and 2. Just like Spacer, GSpacer iteratively computes predecessors (line 10) and blocks them (line 14) in an infinite loop. Whenever a pob is proven to be reachable, the reachable states are updated (line 38). If $Bad$ intersects with a reachable state, GSpacer terminates and returns unsafe  (line 12). If one of the frames is an inductive invariant, GSpacer terminates with safe  (line 20).

When a pob $\langle \varphi , i \rangle$ is handled, we first apply the Concretize rule, if possible (line 7). Recall that Concretize (Algorithm 4) takes as input a cluster that partially blocks $\varphi$ and has a non-linear pattern. To obtain such a cluster, we first find, using $\mathcal {C}_{\textit{pob}}(\langle \varphi , i\rangle )$, a cluster $\langle \pi _1, \mathcal {L}_1 \rangle = \mathcal {C}_{\mathcal {O}_k}(\pi _1)$, where $k \le i$, that includes *some* lemma (from frame *k*) that blocks $\varphi$; if none exists, $\mathcal {L}_1 = \emptyset$. We then filter out from $\mathcal {L}_1$ lemmas that completely block $\varphi$ as well as lemmas that are irrelevant to $\varphi$, i.e., we obtain $\mathcal {L}_2$ by keeping only lemmas that partially block $\varphi$. We apply Concretize on $\langle \pi _1, \mathcal {L}_2\rangle$ to obtain a new pob that under-approximates $\varphi$ if (1) the remaining sub-cluster, $\mathcal {L}_2$, is non-empty, (2) the pattern, $\pi _1$, is non-linear, and (3) $\bigwedge \mathcal {L}_2 \wedge \varphi$ is satisfiable, i.e., a part of $\varphi$ is not blocked by any lemma in $\mathcal {L}_2$.

Once a pob is blocked, and a new lemma that blocks it, $\ell$, is added to the frames, an attempt is made to apply the Subsume and Conjecture rules on a cluster that includes $\ell$. To that end, the function $\mathcal {C}_{\textit{lemma}}(\ell )$ finds *a* cluster $\langle \pi _3, \mathcal {L}_3 \rangle = \mathcal {C}_{\mathcal {O}_i}(\pi _3)$ to which $\ell$ belongs (Sect. 4.2). Note that the choice of cluster is arbitrary. The rules are applied on $\langle \pi _3, \mathcal {L}_3 \rangle$ if the required pre-conditions are met (line 49 and line 53, respectively). When applicable, Subsume returns a new lemma that is added to the frames, while Conjecture returns a new pob that is added to the queue. Note that the latter is a *may* pob, in the sense that some of the states it represents *may not* lead to safety violation.

*Ensuring Progress.* Spacer always makes progress: as its search continues, it establishes absence of counterexamples of deeper and deeper depths. However, GSpacer does not ensure progress. Specifically, unrestricted application of the Concretize and Conjecture rules can make GSpacer diverge even on executions of a fixed bound. In our implementation, we ensure progress by allotting a fixed amount of *gas* to each pattern, $\pi$, that forms a cluster. Each time Concretize or Conjecture is applied to a cluster with $\pi$ as the pattern, $\pi$ loses some gas. Whenever $\pi$ runs out of gas, the rules are no longer applied to any cluster with $\pi$ as the pattern. There are finitely many patterns (assuming LIA terms are normalized). Thus, in each bounded execution of GSpacer, the Concretize and Conjecture rules are applied only a finite number of times, thereby, ensuring progress. Since the Subsume rule does not hinder progress, it is applied without any restriction on gas.

## Evaluation

We have implemented^2^ GSpacer  (Algorithm 5) as an extension to Spacer. To reduce the dimension of a matrix (in subsume, Sect. 4.3), we compute pairwise linear dependencies between all pairs of columns instead of computing the full kernel. This does not necessarily reduce the dimension of the matrix to its rank, but, is sufficient for our benchmarks. We have experimented with computing the full kernel using SageMath [25], but the overall performance did not improve. Clustering is implemented by anti-unification. LIA terms are normalized using default Z3 simplifications. Our implementation also supports global generalization for non-linear CHCs. We have also extended our work to the theory of LRA. We defer the details of this extension to an extended version of the paper.

To evaluate our implementation, we have conducted two sets of experiments^3^. All experiments were run on Intel E5-2690 V2 CPU at 3 GHz with 128 GB memory with a timeout of 10 min. First, to evaluate the performance of local reasoning with global guidance against pure local reasoning, we have compared GSpacer with the latest Spacer, to which we refer as the *baseline*. We took the benchmarks from CHC-COMP 2018 and 2019 [10]. We compare to Spacer because it dominated the competition by solving $85\%$ of the benchmarks in CHC-COMP 2019 ($20\%$ more than the runner up) and $60\%$ of the benchmarks in CHC-COMP 2018 ($10\%$ more than runner up). Our evaluation shows that GSpacer outperforms Spacer both in terms of number of solved instances and, more importantly, in overall robustness.

Second, to examine the performance of local reasoning with global guidance compared to solely global reasoning, we have compared GSpacer with an ML-based data-driven invariant inference tool LinearArbitrary [28]. Compared to other similar approaches, LinearArbitrary stands out by supporting invariants with arbitrary Boolean structure over arbitrary linear predicates. It is completely automated and does not require user-provided predicates, grammars, or any other guidance. For the comparison with LinearArbitrary, we have used both the CHC-COMP benchmarks, as well as the benchmarks from the artifact evaluation of [28]. The machine and timeout remain the same. Our evaluation shows that GSpacer is superior in this case as well.

*Comparison with* Spacer. Table 1 summarizes the comparison between Spacer and GSpacer on CHC-COMP instances. Since both tools can use a variety of interpolation strategies during lemma generalization (Line 45 in Algorithm 5), we compare three different configurations of each: *bw* and *fw* stand for two interpolation strategies, *backward* and *forward*, respectively, already implemented in Spacer, and *sc* stands for turning interpolation off and generalizing lemmas only by *subset clauses* computed by inductive generalization.

**Fig. 2.**
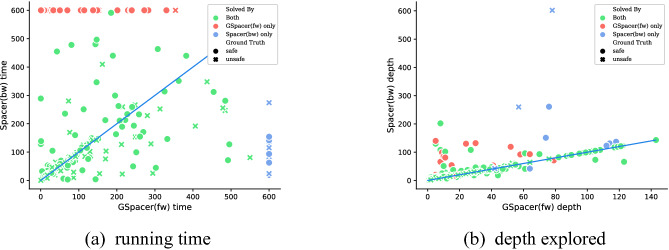
Best configurations: GSpacer versus Spacer.

Any configuration of GSpacer solves significantly more instances than even the best configuration of Spacer. Figure 2 provides a more detailed comparison between the best configurations of both tools in terms of running time and depth of convergence. There is no clear trend in terms of running time on instances solved by both tools. This is not surprising—SMT-solving run time is highly non-deterministic and any change in strategy has a significant impact on performance of SMT queries involved. In terms of depth, it is clear that GSpacer converges at the same or lower depth. The depth is significantly lower for instances solved only by GSpacer.

Moreover, the performance of GSpacer is not significantly affected by the interpolation strategy used. In fact, the configuration *sc* in which interpolation is disabled performs the best in CHC-COMP 2018, and only slightly worse in CHC-COMP 2019! In comparison, disabling interpolation hurts Spacer significantly.

**Fig. 3.**
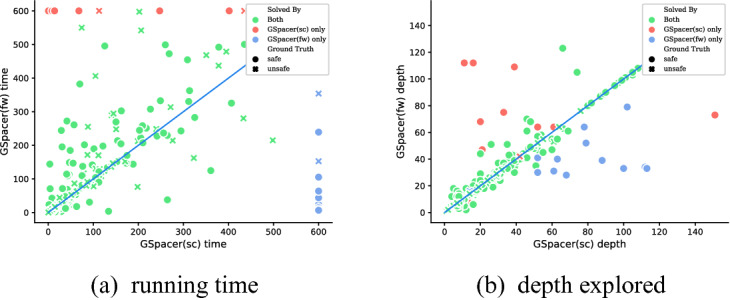
Comparing GSpacer with different interpolation tactics.

Figure 3 provides a detailed comparison of GSpacer with and without interpolation. Interpolation makes no difference to the depth of convergence. This implies that lemmas that are discovered by interpolation are discovered as efficiently by the global rules of GSpacer. On the other hand, interpolation significantly increases the running time. Interestingly, the time spent in interpolation itself is insignificant. However, the lemmas produced by interpolation tend to slow down other aspects of the algorithm. Most of the slow down is in increased time for inductive generalization and in computation of predecessors. The comparison between the other interpolation-enabled strategy and GSpacer (*sc*) shows a similar trend.

**Table 1.**
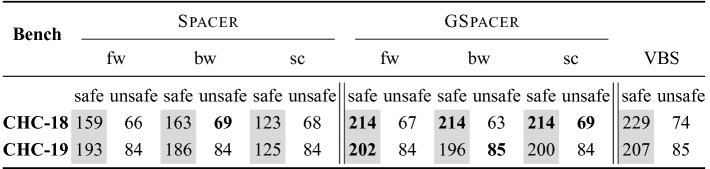
Comparison between Spacer and GSpacer on CHC-COMP.

*Comparison with* LinearArbitrary. In [28], the authors show that LinearArbitrary, to which we refer as LArb for short, significantly outperforms Spacer on a curated subset of benchmarks from SV-COMP [24] competition.

At first, we attempted to compare LArb against GSpacer on the CHC-COMP benchmarks. However, LArb did not perform well on them. Even the baseline Spacer has outperformed LArb significantly. Therefore, for a more meaningful comparison, we have also compared Spacer, LArb and GSpacer on the benchmarks from the artifact evaluation of [28]. The results are summarized in Table 2. As expected, LArb outperforms the baseline Spacer on the safe benchmarks. On unsafe benchmarks, Spacer is significantly better than LArb. In both categories, GSpacer dominates solving more safe benchmarks than either Spacer or LArb, while matching performance of Spacer on unsafe instances. Furthermore, GSpacer remains orders of magnitude faster than LArb on benchmarks that are solved by both. This comparison shows that incorporating local reasoning with global guidance not only mitigates its shortcomings but also surpasses global data-driven reasoning.

**Table 2.**
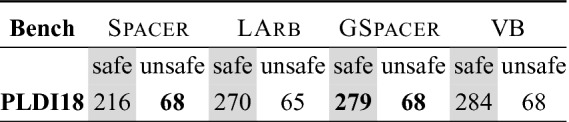
Comparison with LArb.

## Related Work

The limitations of local reasoning in SMT-based infinite state model checking are well known. Most commonly, they are addressed with either (a) different strategies for local generalization in interpolation (e.g., [1, 6, 19, 23]), or (b) shifting the focus to *global* invariant inference by learning an invariant of a restricted shape (e.g., [9, 14–16, 28]).

*Interpolation Strategies.* Albarghouthi and McMillan [1] suggest to minimize the number of literals in an interpolant, arguing that simpler (i.e., fewer half-spaces) interpolants are more likely to generalize. This helps with myopic generalizations (Fig. 1(a)), but not with excessive generalizations (Fig. 1(b)). On the contrary, Blicha et al. [6] decompose interpolants to be numerically simpler (but with more literals), which helps with excessive, but not with myopic, generalizations. Deciding *locally* between these two techniques or on their combination (i.e., some parts of an interpolant might need to be split while others combined) seems impossible. Schindler and Jovanovic [23] propose local interpolation that bounds the number of lemmas generated from a single pob (which helps with Fig. 1(c)), but only if inductive generalization is disabled. Finally, [19] suggests using external guidance, in a form of predicates or terms, to guide interpolation. In contrast, GSpacer uses global guidance, based on the current proof, to direct different local generalization strategies. Thus, the guidance is automatically tuned to the specific instance at hand rather than to a domain of problems.

*Global Invariant Inference.* An alternative to inferring lemmas for the inductive invariant by blocking counterexamples is to enumerate the space of potential candidate invariants [9, 14–16, 28]. This does not suffer from the pitfall of local reasoning. However, it is only effective when the search space is constrained. While these approaches perform well on their target domain, they do not generalize well to a diverse set of benchmarks, as illustrated by results of CHC-COMP and our empirical evaluation in Sect. 5.

*Locality in SMT and* IMC. Local reasoning is also a known issue in SMT, and, in particular, in DPLL(T) (e.g., [22]). However, we are not aware of global guidance techniques for SMT solvers. Interpolation-based Model Checking (IMC) [20, 21] that uses interpolants from proofs, inherits the problem. Compared to IMC, the propagation phase and inductive generalization of IC3 [7], can be seen as providing global guidance using lemmas found in other parts of the search-space. In contrast, GSpacer magnifies such global guidance by exploiting patterns within the lemmas themselves.

*IC3-SMT-based Model Checkers.* There are a number of IC3-style SMT-based infinite state model checkers, including [11, 17, 18]. To our knowledge, none extend the IC3-SMT framework with a global guidance. A rule similar to Subsume is suggested in [26] for the theory of bit-vectors and in [4] for LRA, but in both cases without global guidance. In [4], it is implemented via a combination of syntactic closure with interpolation, whereas we use MBP instead of interpolation. Refinement State Mining in [3] uses similar insights to our Subsume rule to refine predicate abstraction.

## Conclusion and Future Work

This paper introduces *global guidance* to mitigate the limitations of the local reasoning performed by SMT-based IC3-style model checking algorithms. Global guidance is necessary to redirect such algorithms from divergence due to persistent local reasoning. To this end, we present three general rules that introduce new lemmas and pobs by taking a global view of the lemmas learned so far. The new rules are not theory-specific, and, as demonstrated by Algorithm 5, can be incorporated to IC3-style solvers without modifying existing architecture. We instantiate, and implement, the rules for LIA in GSpacer, which extends Spacer.

Our evaluation shows that global guidance brings significant improvements to local reasoning, and surpasses invariant inference based solely on global reasoning. More importantly, global guidance decouples Spacer ’s dependency on interpolation strategy and performs almost equally well under all three interpolation schemes we consider. As such, using global guidance in the context of theories for which no good interpolation procedure exists, with bit-vectors being a primary example, arises as a promising direction for future research.

## Footnotes

1 In fact, a primed copy is introduced in $\varSigma '$ only for the uninterpreted symbols in $\varSigma$. Interpreted symbols remain the same in $\varSigma '$.

2 https://github.com/hgvk94/z3/tree/gspacer-cav-ae.

3 Detailed experimental results including the effectiveness of each rule, and the extensions to non-linear CHCs and LRA can be found at https://hgvk94.github.io/gspacer/.
